# Does Raising Type 1 Error Rate Improve Power to Detect Interactions in Linear Regression Models? A Simulation Study

**DOI:** 10.1371/journal.pone.0071079

**Published:** 2013-08-16

**Authors:** Casey P. Durand

**Affiliations:** Michael & Susan Dell Center for Healthy Living, Division of Health Promotion and Behavioral Sciences, University of Texas School of Public Health, Houston, Texas, United States of America; Sapienza University of Rome, Italy

## Abstract

**Introduction:**

Statistical interactions are a common component of data analysis across a broad range of scientific disciplines. However, the statistical power to detect interactions is often undesirably low. One solution is to elevate the Type 1 error rate so that important interactions are not missed in a low power situation. To date, no study has quantified the effects of this practice on power in a linear regression model.

**Methods:**

A Monte Carlo simulation study was performed. A continuous dependent variable was specified, along with three types of interactions: continuous variable by continuous variable; continuous by dichotomous; and dichotomous by dichotomous. For each of the three scenarios, the interaction effect sizes, sample sizes, and Type 1 error rate were varied, resulting in a total of 240 unique simulations.

**Results:**

In general, power to detect the interaction effect was either so low or so high at α = 0.05 that raising the Type 1 error rate only served to increase the probability of including a spurious interaction in the model. A small number of scenarios were identified in which an elevated Type 1 error rate may be justified.

**Conclusions:**

Routinely elevating Type 1 error rate when testing interaction effects is not an advisable practice. Researchers are best served by positing interaction effects *a priori* and accounting for them when conducting sample size calculations.

## Introduction

Interaction effects are an important component of statistical analysis in many scientific disciplines. Interactions, also referred to as moderation or effect modification, answer the question of whether the relationship between a predictor and outcome variable depends on the value of some third variable [1], [2]. As an example, an epidemiologist may want to know whether the effect of physical activity on body mass index (BMI) varies by age, with age serving as the moderator. In this case, the two predictor variables are continuous, but the effect can be generalized to other cases, such as when one or both of the predictors are categorical. An example of the latter would be when seeking to understand whether the effect of statin use on cholesterol level varies depending on whether someone is male or female. Thus, the value of interactions lies in their ability to provide a more nuanced picture of the primary relationship under investigation.

In a linear regression model, a basic interaction may be expressed using the following form:
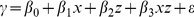
(1)In this equation, 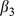 represents the effect of the variable 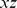 on 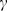; 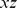 is the product of variables 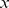 and 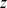. Variable 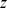 is said to moderate the relationship between 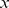 and 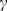 when 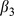 is statistically significant [1], [2]. We may alternatively say that variable 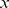 moderates the relationship between 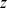 and 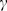. Whether variable 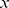 or 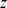 serves as the moderator in equation 1 should be determined *a priori*; there is no statistical difference in selecting one or the other. This basic model can be extended to include multiple interactions or interactions involving three or more variables.

Because the focus is on statistical significance, the ability to detect interactions is intimately tied to the power of that test. Power is the probability of rejecting a false null hypothesis. Prior research has shown that statistical power to detect interactions is lower than for main effects (i.e., the relationship between 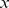 and 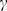, and 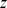 and 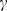) for a variety of reasons. For example, unreliability of measurements (i.e. measurement error) is widely known to weaken the observed association between an independent and dependent variable. This is compounded in the interaction term, since its reliability is the product of the reliability of the lower order variables [3], [4]. Also, when study participants are not representative of the underlying population due to nonrandom sampling, the variance of measures may be reduced relative to the true level in the larger population [3]–[5]. The restricted variance of measures makes it more difficult to identify significant interactions, since like measurement error it is compounded in the interaction term. Other reasons power to detect interaction effects is lower include uneven sub-group sample sizes when one or both of the predictors involved in the interaction term is categorical and the fact that interaction effect sizes are likely smaller than main effect sizes, since it is well known that smaller effects require larger sample sizes to detect [2]–[6].

The decrease in power to detect interaction effects can be quite dramatic. A study by Brookes et al simulated the power to detect interaction effects (i.e. subgroup effects) in clinical trials [7]. As with many other analyses, clinical trials are frequently not powered *a priori* to detect interactions. For a trial powered at 80% to detect main effects, the power to detect an interaction effect the same size as the main effect was only 29%. For an interaction effect one-half the size of the main effect, power was closer to 10%. A trial's sample size would have to be increased by a factor of 16 in order to have power to detect an interaction equivalent to the power to detect the main effect, assuming an interaction effect size is one-half as large as the main effect size. This study makes clear that underpowered interaction tests pose a serious problem, greatly increasing the risk of incorrectly concluding that no interaction effect exists when in reality it does.

The best recommendation for dealing with decreased power to detect statistical interactions is to plan for interaction testing during the study development phase, and based on expected effect sizes and other parameters noted above, collect data on a sufficient number of observations to have the desired power to detect a true effect [2]. Of course, sample size calculations may indicate the need for more participants than one has the resources to collect data on. Further, new questions involving interactions not foreseen by the study planners may arise in the course of secondary analysis of an existing dataset. The original design may only have been sufficiently powered to detect main effects.

So for these reasons, a researcher may be forced to deal with insufficient power to detect an interaction with no way to add more observations. What then, are they to do? One approach is to simply ignore the issue of power and proceed as planned. However, this risks missing an important true interaction simply due to low power. An alternative approach is to raise the Type 1 error rate [2], [3], [8]. This approach has been recommended for both primary and secondary research, such as when conducting statistical tests of heterogeneity in a meta-analysis [9]. Type 1 error refers to incorrectly rejecting a true null hypothesis, and is linked to significance levels (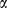) in that the latter is the long-run probability of making a Type 1 error (i.e. Type 1 error rate). Due to the relationship between Type 1 error and power, utilizing a higher Type 1 error rate necessarily results in increased power. Though it does not appear to be standard practice, it is not unusual to find studies following this procedure, in some cases elevating the Type 1 error rate to as high as 20% [10]–[13]. This approach represents a tradeoff in which researchers are increasing the probability of detecting a true interaction, while simultaneously increasing the probability of accepting an interaction that is in reality spurious. The theoretical importance of detecting an interaction may be sufficient to justify that tradeoff. While the researcher must ultimately make this decision, balancing Type 1 error and power may be best suited for exploratory work where the aim is to generate new hypotheses for future study, rather than for something like an efficacy trial, where the goal is not so much to generate hypotheses as test and confirm or disconfirm them [14].

However, as Marshall has pointed out, raising Type 1 error rate to increase power can be problematic, depending on what the current level of power is at the nominal level of significance [8]. If a study already has sufficient power to detect an interaction, then raising Type 1 error rate will do nothing other than increase the likelihood that inconsequential interactions are accepted and incorporated into the statistical model. This could be especially problematic in large models with many predictors, and thus many possible two-way, three-way, etc. interactions. Conversely, if power at nominal levels is already compromised, then raising Type 1 error rate may only make a bad situation slightly better, though still not sufficient, at the cost of a higher probability of committing Type 1 errors.

Despite this, there may be a middle ground in which raising the Type 1 error rate results in an acceptable tradeoff between increased statistical power and higher Type 1 error rates. These would be samples that are marginally powered to detect an interaction at nominal significance levels; boosting that level may provide a meaningful gain in power to detect what the researcher believes to be an important interaction effect.

Marshall has previously conducted an analysis in which he identified these three scenarios for case-control logistic regression analyses in order to provide a rough guide as to which situations might warrant raising Type 1 error rates [8]. Given the commonplace role of linear regression estimated using ordinary least squares in data analysis, researchers may find it useful to understand the equivalent scenarios for these models. Therefore the aim of this paper is to identify which combinations of variable types, effect sizes and sample sizes warrant consideration of increased Type 1 error rates in order to have sufficient power to detect a statistical interaction in a linear regression model with a continuous dependent variable.

## Methods

Power was examined using a Monte Carlo simulation. By repeatedly generating and analyzing numerous samples of data, a simulation can provide insight into the behavior of estimators or statistics of interest in finite samples. A simulation study follows several steps [15], [16]. First, we generate a “population” of a specified size. Second, for this population, we generate two variables 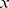 and 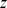 drawn randomly from the standard normal distribution, and compute their product to form the interaction term 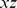. Third, we generate an error term 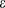 drawn randomly from the standard normal distribution representing the stochastic component of the regression model. This error is the difference between the average value of 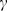 in the population with given values of 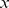 and 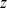 and an individual's true value of the dependent variable, assuming the same values of 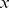 and 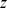. Fourth, a true dependent variable 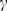 is generated using formula 1, where 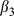 is the difference in slope between two levels or values of the moderating variable. That is, 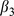 represents the effect size of the interaction we seek to detect; 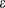 is the stochastic error generated by the previous step. Fifth, we regress true 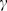 on 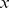, 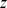 and 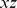 and store the p-value associated with the interaction term. We repeat the last three steps for a total of 10,000 replications. The percentage of p-values that are less than our *a priori* Type 1 error rate is our empirical power, because we have constructed our synthetic dataset in such a way that there is truly a non-zero relationship between 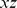 and 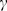. Recall that power is the probability of rejecting a false or incorrect null hypothesis. It should be noted that analytic equations exist for calculating power (or sample size) in this context, and depending on circumstances may be more straightforward than programming a simulation. Interested readers should see Dupont and Plummer (1998) [17]. For the scenarios considered here, the analytic and simulation approaches yielded largely equivalent results, particularly with larger sample sizes.

We vary the Type 1 error rate, sample size, independent variable type, and effect size associated with the interaction term, resulting in a total of 240 distinct simulations. The exact parameter values used can be seen in table 1. The values of 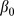,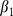 and 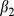
are set to 1, without loss of generality. The data generating process assumes that all variables are measured without error and the predictors and the random error are uncorrelated with each other. In constructing the interaction terms, three types of variable combinations were examined: continuous by continuous, continuous by dichotomous, and dichotomous by dichotomous. Continuous variables were specified to follow the standard normal distribution, while dichotomous variables were also standard normal variables subsequently dichotomized such that there was an approximately equal split of the sample into each category. Our choice of effect sizes are derived from a simulation study of power to detect mediation effects by Fritz and MacKinnon, who in turn based their effect sizes on Cohen's small, medium and large effect size criteria [18]. It should be noted that our use of standardized coefficients in this simulation study does not constitute an endorsement of their use in practice. They have been criticized for many reasons, and without careful attention will result in difficult-to-interpret interaction effects when generated automatically by statistical software packages [1], [19]–[21]. We use them here because they provide a way to make our simulation results as generally applicable as possible. Whether an effect size is large or small should ultimately be based on a careful understanding of the substantive area of research.

**Table 1:** Parameters set to vary across simulations.

Parameter	Varying Values
Type 1 error rate	5%, 10%, 15%, 20%
Sample size	50, 200, 300, 500, 1000
Interaction effect size (β_3_)	0.05, 0.14, 0.26, 0.39
Interaction variable combinations	Continuous by continuous; Continuous by dichotomous; Dichotomous by dichotomous

Following the procedures of Marshall, we defined a useful gain in power (i.e. a gain in power sufficient to justify the higher Type 1 error rate) as a relative increase in power of at least 10% when going from a 5% to 20% Type 1 error rate; power had to be at least 80% at the 20% error rate. Power of 80% was chosen to be consistent with research norms; this differs from Marshall, who required power of 70%. The 10% relative increase requirement was chosen to avoid having interactions marginally powered at the 5% Type 1 error rate (e.g. 75% power) that subsequently achieve 80% or slightly higher power at the 20% error rate be classified as achieving a useful gain. Such a small gain, while ultimately achieving 80% power, is likely not sufficient to justify the risk associated with the elevated Type 1 error rate. Analyses were conducted in Stata 12.1 (StataCorp, College Station, TX) based on methods outlined by Feivson (2002) and Arnold (2011). Complete Stata syntax to run the simulations is available from the author upon request.

## Results

As a check on the accuracy of our simulations, we first ran models in which the effect size for the interaction term was set to zero. If the simulation is functioning properly, then the estimated power should be equivalent to the Type 1 error rate, within sampling error. All results met this criterion, verifying the simulation procedures (data not shown).

### Continuous by continuous

**Figure 1:**
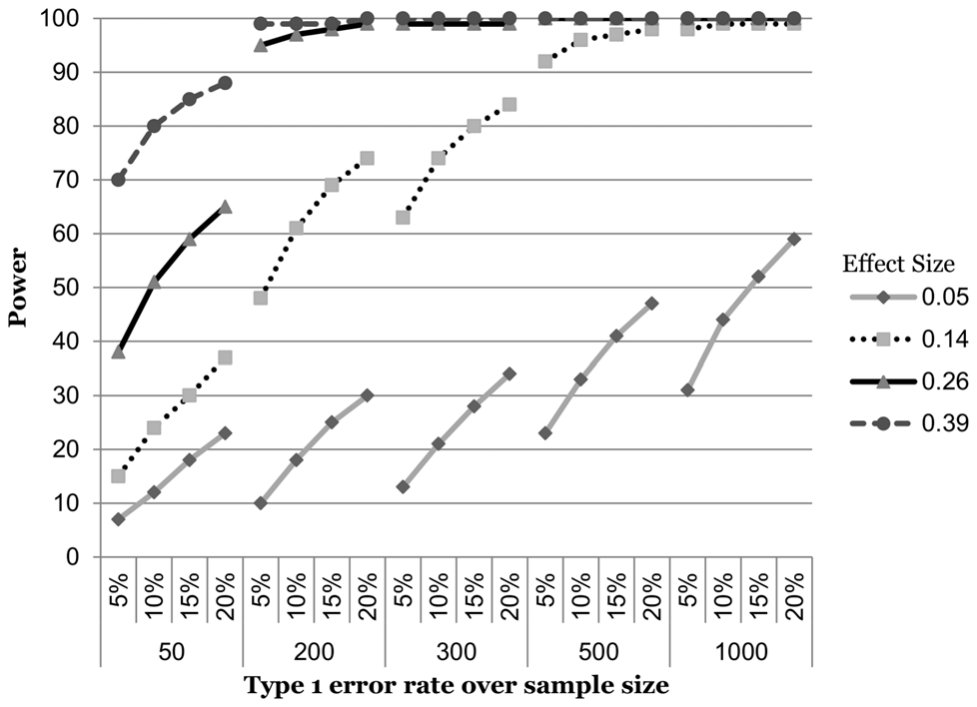
Power curves for an interaction of two continuous variables.

Monte Carlo simulation results for the continuous by continuous interaction are shown in Figure 1. Only two combinations of effect and sample sizes showed any promise for elevating the Type 1 error rate from 5% to 20%. At an effect size of 0.39 and sample size of 50, power increased by 26%. However, 80% power could be achieved by raising the error rate to only 10%. At an effect size of 0.14 and sample size of 300, power increased by 33%. Power of 80% could be achieved by raising the error rate to 15%. Of the remaining 18 sample and effect size combinations, eight never achieved acceptable power, while 10 were already sufficiently powered at a Type 1 error rate of 5%.

### Continuous by dichotomous

**Figure 2:**
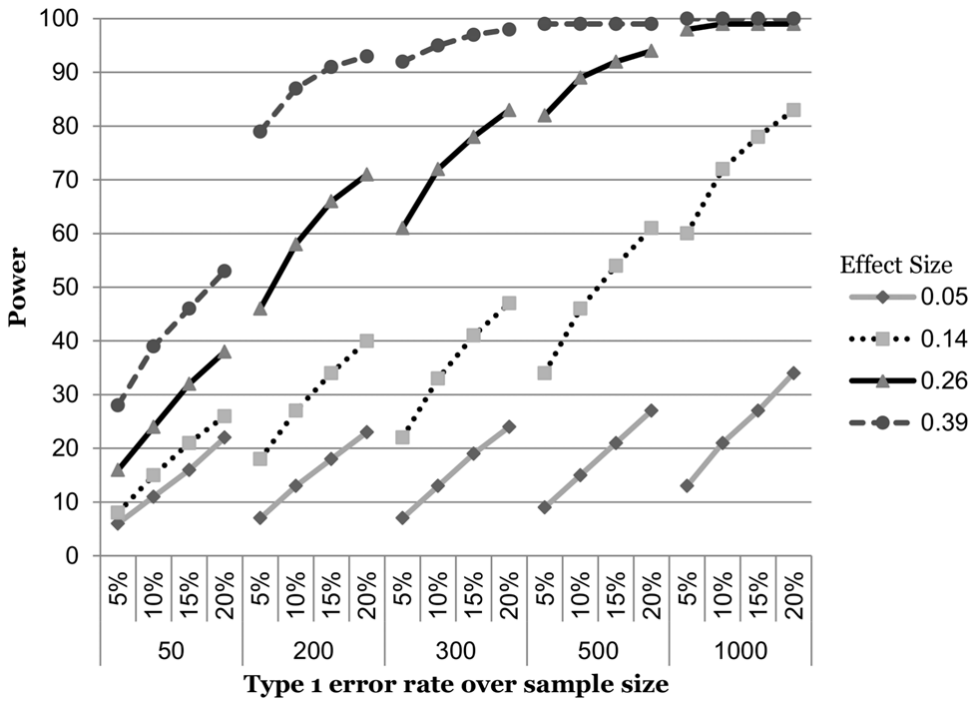
Power curves for an interaction of continuous and dichotomous variables.

For the continuous by dichotomous interactions (Figure 2), three effect and sample size combinations appeared to justify the increase in Type 1 error rate. At an effect size of 0.39 and sample of 200, power increased by 18%, though this was almost sufficiently powered (79%) at the 5% level. At a sample size of 300 and effect size of 0.26, power increased by 36%. A similar increase in power was noted for a sample size of 1000 and effect size of 0.14. Of the 17 remaining sample and effect size combinations, 12 never achieved sufficient power, and five had adequate power at the 5% level.

### Dichotomous by dichotomous

**Figure 3:**
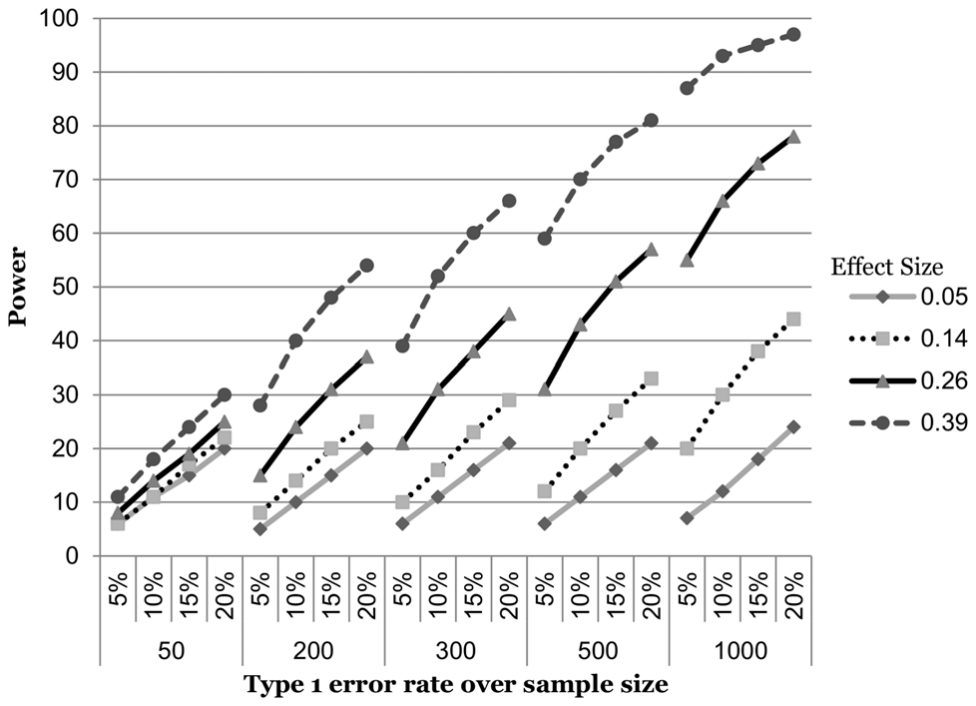
Power curves for an interaction of two dichotomous variables.

Finally, for the dichotomous by dichotomous interactions (Figure 3), only for the sample size of 500 and effect size of 0.39 was there a useful gain in power of 37%. For the 19 remaining combinations, 18 never achieved sufficient power, while one was sufficiently powered at 5%.

## Discussion

The goal of this analysis was to determine whether there were any situations in which raising the Type 1 error rate would lead to a useful gain in power to detect interaction effects in linear regression models. Of the 60 combinations of interaction type, effect size and sample size, we found only six scenarios that could be called the “middle ground” mentioned in the introduction, in which heightened ability to detect an effect may justify the risk assumed when elevating the Type 1 error rate. For the most part, power was either so poor or so good at the 5% level that raising the error rate made no meaningful difference besides increasing the possibility that spurious interactions would be accepted. As Marshall found for logistic regression models, so too do we find that elevating the Type 1 error rate as a routine matter of practice when testing interactions is probably not advisable. Researchers would be best served by taking these results as a general guide, followed up with power calculations specific to their sample size, expected effect size, and the other factors known to impact power. Only then can an informed decision be made about whether increasing the Type 1 error rate is justified.

Two other important points should be made. First, given how low power was, even at an elevated level of statistical significance, for many scenarios, a researcher's failure to detect an interaction effect does not necessarily mean the effect does not exist [3]. The test simply may have lacked power. While this statement is true for any probabilistic test, it is particularly true for interactions, since, as noted in the introduction, power is lower for interactions than main effects to begin with. Therefore, under one of the many low power scenarios, interactions may still be incorporated into models despite statistical insignificance if there is strong *a priori* reason stemming from theory or previous research to include them.

Second, it is interesting to note how severely power is compromised when the interaction term is made up of one or two dichotomous variables as compared to when both variables are continuous. This can be seen in the shift down and to the right in the power curves for the latter two interaction types (figures 2 & 3). This should not be surprising, as many authors have noted the loss in power (among other problems) associated with turning a naturally continuous variable into a categorical one [6], [22]–[24]. Under the two dichotomous variable scenarios, power is so diminished that even the elevated Type 1 error rate does nothing to help except for the very largest effect size in the sample size of 500. Our results serve as yet another reminder that under most circumstances, continuous variables should remain that way.

### Strengths

A major strength of this paper is the range of scenarios we considered. By examining different combinations of variable types, effect sizes, sample sizes, and Type 1 error rate, we have captured numerous situations that applied researchers may expect to encounter, thus increasing the usefulness of our results. Another strength is the use of a Monte Carlo simulation study. Instead of relying on the asymptotic properties of formulas to calculate power, we have determined it empirically. This is especially useful given the small sample sizes of some of the scenarios we have considered.

### Limitations

While we have examined many interaction scenarios, our treatment was not comprehensive. We did not include any three-way or higher order interactions, more than one interaction per model, categorical variables with more than 2 categories, or ordinal variables. By necessity, we made many assumptions about the data generating process, including that all continuous variables were normally distributed, dichotomous variables were a perfect 50–50 split, and predictor variables were uncorrelated and measured without error. Deviations from this specification could impact power. Finally, our criteria for a useful gain in power were subjective and may not be appropriate in all situations; different criteria may result in different conclusions.

## Conclusions

When testing interaction effects in linear regression models, there are likely few circumstances in which raising the Type 1 error rate results in a gain in power that may be sufficient to justify the potential acceptance of spurious effects. While power is always best addressed prior to undertaking a study, researchers utilizing existing datasets may use these results to guide a thoughtful discussion of whether it is appropriate to use an elevated Type 1 error rate to test interactions in their own statistical models.

## References

[1] Whisman MA, McClelland GH (2005) Designing, testing, and interpreting interactions and moderator effects in family research. J Fam Psychol 19: 111–120. 15796657 10.1037/0893-3200.19.1.111

[2] McClelland GH, Judd CM (1993) Statistical difficulties of detecting interactions and moderator effects. Psychol Bull114: 376–390. 10.1037/0033-2909.114.2.376 8416037

[3] Aguinis H (1995) Statistical power problems with moderated multiple regression in management research. J Manage 21: 1141–1158.

[4] Aguinis H, Boik RJ, Pierce CA (2001) A generalized solution for approximating the power to detect effects of categorical moderator variables using multiple regression. Organ Res Methods 4: 291–323.

[5] Aguinis H, Stone-Romero EF (1997) Methodological artifacts in moderated multiple regression and their effects on statistical power. J Appl Psychol 82: 192–205.

[6] Stone-Romero EF, Alliger GM, Aguinis H (1994) Type II error problems in the use of moderated multiple regression for the detection of moderating effects of dichotomous variables. J Manage 20: 167–178.

[7] Brookes ST, Whitely E, Egger M, Smith GD, Mulheran PA, et al (2004) Subgroup analyses in randomized trials: Risks of subgroup-specific analyses;: Power and sample size for the interaction test. J Clin Epidemiol 57: 229–236. 15066682 10.1016/j.jclinepi.2003.08.009

[8] Marshall S (2007) Power for tests of interaction: Effect of raising the type I error rate. Epidemiol Perspect Innov 4: 4. 17578572 10.1186/1742-5573-4-4 PMC1910596

[9] Petitti DB (2001) Approaches to heterogeneity in meta-analysis. Stat Med 20: 3625–3633. 11746342 10.1002/sim.1091

[10] Fagherazzi G, Vilier A, Balkau B, Clavel-Chapelon F, Magliano DJ (2013) Anthropometrics, body shape over 12 years and risk of cancer events in pre- and post-menopausal women. Int J Cancer: In press. 10.1002/ijc.28069 23364907

[11] Olivier J, Walter SR, Grzebieta RH (2013) Long term bicycle related head injury trends for new south wales, australia following mandatory helmet legislation. Accid Anal Prev 50: 1128–1134. 23026203 10.1016/j.aap.2012.09.003

[12] Choi B, Ostergren P, Canivet C, Moghadassi M, Lindeberg S, et al (2011) Synergistic interaction effect between job control and social support at work on general psychological distress. Int Arch Occup Environ Health 84: 77–89. 20582551 10.1007/s00420-010-0554-y PMC3016236

[13] Kendall E, Terry D (2009) Predicting emotional well-being following traumatic brain injury: A test of mediated and moderated models. Soc Sci Med 69: 947–954. 19616354 10.1016/j.socscimed.2009.06.021

[14] Moye LA (2003) Multiple analyses in clinical trials: Fundamentals for investigators. New York: Springer.

[15] Feivson AH (2002) Power by simulation. Stata J 2: 107–124.

[16] Arnold B, Hogan D, Colford J, Hubbard A (2011) Simulation methods to estimate design power: An overview for applied research. BMC Med Res Methodol11: 94. 10.1186/1471-2288-11-94 PMC3146952 21689447

[17] Dupont WD, Plummer WD (1998) Power and sample size calculations for studies involving linear regression. Control Clin Trials 19: 589–601. 9875838 10.1016/s0197-2456(98)00037-3

[18] Fritz MSM, David P (2007) Required sample size to detect the mediated effect. Psychol Sci 18: 233–239. 17444920 10.1111/j.1467-9280.2007.01882.x PMC2843527

[19] King G (1986) How not to lie with statistics: Avoiding common mistakes in quantitative political science. J Polit Sci 30: 666–687.

[20] Blalock HM (1961) Evaluating the relative importance of variables. Am Sociol Rev 26: 866–874.

[21] Greenland S, Schlesselman JJ, Criqui MH (1986) The fallacy of employing standardized regression coefficients and correlations as measures of effect. Am J Epidemiol 123: 203–208. 3946370 10.1093/oxfordjournals.aje.a114229

[22] MacCallum RC, Zhang S, Preacher KJ, Rucker DD (2002) On the practice of dichotomization of quantitative variables. Psychol Methods7: 19–40. 10.1037/1082-989x.7.1.19 11928888

[23] Royston P, Altman DG, Sauerbrei W (2006) Dichotomizing continuous predictors in multiple regression: A bad idea. Stat Med 25: 127–141. 16217841 10.1002/sim.2331

[24] Bennette C, Vickers A (2012) Against quantiles: Categorization of continuous variables in epidemiologic research, and its discontents. BMC Med Res Methodol 12: 21. 22375553 10.1186/1471-2288-12-21 PMC3353173

